# Effects of weight divisions in time-motion of female high-level Brazilian Jiu-jitsu combat behaviors

**DOI:** 10.3389/fpsyg.2023.1048642

**Published:** 2023-02-13

**Authors:** Marco Antonio Ferreira dos Santos, Dany Alexis Sobarzo Soto, Michele Andrade de Brito, Ciro José Brito, Esteban Aedo-Muñoz, Maamer Slimani, Nicola L. Bragazzi, Hela Znazen, Bianca Miarka

**Affiliations:** ^1^Laboratory of Psychophysiology and Performance in Sports and Combats, Postgraduate Program in Physical Education, Federal University of Rio de Janeiro, Rio de Janeiro, Brazil; ^2^Escuela de Kinesiología, Facultad de Salud, Universidad Santo Tomás, Puerto Montt, Chile; ^3^Postgraduate Program in Physical Education, Federal University of Juiz de Fora, Governador Valadares, Brazil; ^4^Departamento de Educación Física, Deportes y Recreación, Facultad de Artes y Educación Física, Universidad Metropolitana de Ciencias de la Educación,Santiago, Chile; ^5^Department of Health Sciences (DISSAL), University of Genoa, Genoa, Italy; ^6^Department of Mathematics and Statistics, Laboratory for Industrial and Applied Mathematics (LIAM), York University, Toronto, ON, Canada; ^7^Department of Physical Education and Sport, College of Education, Taif University, Taif, Saudi Arabia

**Keywords:** sports psychology, technical-tactical analysis, task performance and analysis, judo, martial arts

## Abstract

Coaches and psychologists can use time-motion analysis to elaborate specific interventions for female BJJ athletes, increasing specific training context and reducing unnecessary psychological and physical demands and injuries. Therefore, the present study aimed to analyze high-level BJJ female athletes in the 2020 Pan-American Games by comparing the weight categories on the time-motion analysis. The time-motion analysis (i.e., approach, gripping, attack, defensive actions, transition, mounting, guard, side control, and submissions) of 422 high-level female BJJ combats was divided and compared by weight category as follows: Rooster (*n* = 8), Light Feather (*n* = 18), Feather (*n* = 122), Light (*n* = 84), Middle (*n* = 74), Medium Heavy (*n* = 44), Heavy (*n* = 36), Super Heavy (*n* = 36), using *p* ≤ 0.05. The main results indicated that the Super heavyweight category [3.1 (5.8;119.9) s] had a shorter gripping time than other weight categories, *p* ≤ 0.05. In contrast, roosters [7.2 (3.5;64.6) s] had longer gripping, transition [14.0 (4.8;29.6) s], and attack time [76.2 (27.7, 93.2)] than the light feather, middlers, and heavier weight categories, *p* ≤ 0.05. These findings should be considered for the psychological interventions and training prescription.

## 1. Introduction

Female Brazilian Jiu-jitsu (BJJ) practitioners are growing, and the demand for scientific studies generating gender-specific training approaches is needed. An inclusive understanding of the time-motion combat demands of female combat athletes could positively impact the success rate and help in training efficiency (Williams et al., 2019; Ambroży et al., 2021; Pawelec et al., 2022). The performance in grappling combat sports is related to the ability of female or male competitors to execute specific actions at the right moment during each combat phase while quickly adjusting to the continually changing combat context to the following action or phase (Sterkowicz-Przybycień et al., 2017). To the best of our knowledge, no study compared the weight categories of female Brazilian jiu-jitsu athletes in the phases of approach, gripping, attack, defensive actions, and pause. Coaches and psychologists can use the evidence offered here to elaborate specific interventions for female BJJ athletes, increasing specific training contexts and reducing unnecessary psychological and physical demands and injuries (Andreato et al., 2015; Brandt et al., 2021; Santos et al., 2022). Time-motion schemes can be developed that stimulate the acquisition of skills, and at the same time, the athlete has to neutralize opponents’ strategies (Miarka et al., 2017b, 2020c, Menescardi et al., 2020).

The time-motion analysis per combat cycle identifies action patterns, often physiological inferences, in the competitive situation of female grappling athletes (Challis et al., 2015; Dudeniene et al., 2017; Soriano et al., 2019; Menescardi et al., 2020). The time-motion analysis can help BJJ coaches and athletes develop specific training for adequately applying the approach, gripping, attack, defense, transition, submission, and all other movements during the combats (Coswig et al., 2018a,b). Investigations with grappling athletes have shown that some of these variables present differentiated frequencies in weight categories (Sterkowicz-Przybycień et al., 2017; Soto et al., 2020a; Dopico-Calvo et al., 2022; Miarka et al., 2022). In a preliminary study, female senior grapplers had longer total combat time, standing combat time, and gripping time than pre-cadet, cadet, and junior athletes (Miarka et al., 2014). Challis et al. (2015) showed work-to-rest ratios of 3:1 blocks off lightweight women’s judo matches. Olympic and non-Olympic judo matches by competitive result (winning versus losing) demonstrated differences in decision-making, gripping frequencies, and attack orientations of female athletes (Miarka et al., 2016b). Despite some motor skills similarities between Judo and BJJ, the rules are very different, and no study compared weight categories in the phases of approach, gripping, attack, defensive, groundwork, and pause, verifying the effects of weight categories of female BJJ high-level athletes.

Time-motion analysis identified action and moment patterns of different weight categories in other combat sports studies (Slimani et al., 2017; Soto et al., 2020b; Sciranka et al., 2022). Initially, the authors indicated that the time-motion analysis presents convenient results to the BJJ athletes’ training process, suggesting the observation by the coaches of the metabolic components and muscle power. Another study (Del Vecchio et al., 2011) demonstrated the importance of the effort-pause relationship phases in male BJJ matches. Other authors indicated that type of analysis allows observing how athletes tactically use energy systems according to the dynamics of grappling combats (Andreato et al., 2013, 2015; Coswig et al., 2018b; Barreto et al., 2021) classifying their structure in effort-pause, high-intensity, and low-intensity actions during BJJ combat. Conceptually, high-intensity actions occur when the athlete attempts to advance, progress, or evolve with strength or power (Carvalho et al., 2022a). In contrast, low-intensity actions are classified as actions in which movements are performed slowly, with an apparent reduced level of force and power application (Coswig et al., 2018b).

For instance, Coswig et al. (2018a) demonstrate that a male BJJ match has an average of 8.5 high-intensity blocks, 17.9 low-intensity blocks, and eight pause blocks, indicating that the effort-pause ratio is 22:1 and the high-intensity versus low-intensity ratio is of 1:3.5. Authors also indicated that the proportion of effort in a BJJ match is more significant than 90% and that the BJJ athlete spends most of the groundwork phase (Coswig et al., 2018b). Therefore, when investigating the total time of actions, the study results that high-intensity total time is on average 226 s, low intensity is 303 s, with an average pause time of 71 s (Coswig et al., 2018b). This research also showed that concerning high-intensity blocks, the percentage was 37 and 50% for low-intensity blocks, having found 31 high-intensity blocks and 26 low-intensity blocks, generating the effort-pause relationship. 8: 1 and the high and low-intensity ratio at 1:2 (Coswig et al., 2018b).

Andreato et al. (2013) observed in male BJJ combats that the effort time was 296 s, and each block had 117 s of effort, with an effort of 2 blocks. The total time of pauses was 33 s, with an average of 20 s of pauses per block (Andreato et al., 2013). The total high-intensity time was 24 s, with an average of 3 s per high-intensity block, adding up to an average of 8 high-intensity blocks. The total low-intensity effort was 249 s on average, with 25 s on average per block, containing nine blocks and low intensity (Andreato et al., 2013). In a subsequent study, when comparing four bouts of 10 min, the average effort was found in 250 s in the first BJJ fight, with three blocks of effort, 180 s in the second fight, with four blocks of effort, 290 s on average, containing three blocks of effort. In the fourth fight, the authors found 204 s, on average, containing three blocks of effort (Andreato et al., 2015).

Regarding pauses, the result of this study pointed out that in the first combat, the pause was 32 s with two blocks, in the second fight, it got 26 s on average, with three pause blocks, and in the third fight, it found an average of 21 s of pause, with two blocks. In the fourth fight, the average time was 44 s, with two blocks of pauses on average. Finally, the effort-pause ratio was characterized as follows: first fight 8:1, second fight 9:1, third fight 8:1, and fourth fight 6:1 (Andreato et al., 2015).

Female athletes can use the evidence presented here to elaborate on specific BJJ training for each weight category if there are differences between groups (Miarka et al., 2017b; Blach et al., 2021). In addition, strategies can be developed that stimulate skills acquisition and avoid injuries (Santos et al., 2022; Carvalho et al., 2022b). At the same time, the athlete can use this strategy to neutralize the strategy of opponents. Therefore, the present study aimed to analyze high-level BJJ female athletes in the 2020 Pan-American Games by comparing the weight categories on the time-motion analysis (i.e., approach, gripping, attack, defensive actions, transition, mounting, guard, side control, and submissions).

## 2. Methods

### 2.1. Sample

The sample number consisted of 422 female match analyses, representing the total number of female fights at the 2020 BJJ Pan American (Kissimmee, Florida, EUA) of the International Brazilian Jiu-Jitsu Federation - BJJF. The sample was divided by weight category as follows: Rooster (*n* = 8), Light Feather (*n* = 18), Feather (*n* = 122), Light (*n* = 84), Middle (*n* = 74), Medium Heavy (*n* = 44), Heavy (*n* = 36), Super Heavy (*n* = 36) and correlated with time-movement analysis in the phases of approach, grip, transition, guard, lateral control, mount, attack, defensive actions, and movement expressed in seconds.

The sample calculation representing female international combats obtained a 99% confidence level and 1% margin of error, using the equation below (Jill, 2010):

*n* = *N Z*^2^ *p* (1-*p*) (*N*–1) *e*^2^ + *Z*^2^ *p* (1-*p*).

The interpretation of each of these elements was made as follows:

*n* = is the sample size obtained through the calculation;

*N* = total combats belonging to the championship;

*Z* = indicated deviation from the acceptable mean value for the confidence level to be reached;

*e* = is the maximum margin of error that the search allows;

*p* = is the proportion we want to find in the calculation.

All data used for analysis were taken from a public domain website.^1^ As public data, the present study was released from the local Research Ethics Committee following the WMA Declaration of Helsinki.

### 2.2. Procedures and measurements

The protocol variables of this study were divided into a macro group that allowed the grouping of the techniques used. When classifying a particular technique, the analyst had to inform which group the technique belonged to, then define the technique, and then choose the laterality of the technique’s application, as well as inform whether this technique generated points or submission.

The instrument of this study considers the phases of BJJ combat according to a previous protocol of Frami software (Miarka et al., 2011; Barrientos et al., 2021; i.e., approach, gripping, attacks, defense, movement), expanded by the present study (transition, control side, guard, and mounted) taking into account the specifics of BJJ combats.

Approach time: Non-contact displacements, when athletes remain for a few seconds observing the opposing actions without performing any contact action. Or a specific location on the opposing athlete’s kimono (Brito et al., 2017; Barreto et al., 2019; Soto et al., 2020b).

Gripping time: When starting a BJJ contact between athletes, the gripping stage is a relevant motor action. It is the ability to perform and maintain the grip (handgrip) in the opponent’s kimono (Miarka et al., 2016a; Dal Bello et al., 2019).

Transition time: One of the techniques that appear in the combat’s initial moments are the projections or throws, which can result in a score for the athlete who executes efficiently. However, these techniques have lost space for the guard pull. A variety of techniques is used to lead the combat to the ground without the risk of making a throw that can be defended by the opponent (Sterkowicz-Przybycień et al., 2017; Coswig et al., 2018a).

Guard time: A guard is a groundwork position where an athlete wraps his/her/it legs around the opponent, restricting movement and forcing contact, trying to prevent the adversary inside the guard from standing up or escaping (Del Vecchio et al., 2016; Lima et al., 2017).

Side control time: Side control is one of the most frequent positions on the groundwork that allows athletes to control and submit to their opponents (Kirk et al., 2015; Miarka et al., 2020c). It happens when the athlete lays perpendicularly on top of his adversary (Kirk et al., 2015; Miarka et al., 2020c).

Mounting time: This action is a submission where the athlete is on top of his/her/it opponent and facing their head (Kirk et al., 2015). Athletes sit on top of the adversary’s torso in a kneeling-like configuration, and his/her/it hips are over his/her/it torso. Athletes’ weight should be primarily on the opponent’s body to make it difficult for them to move (Kirk et al., 2015).

Attack and Defense Time: The principal techniques grouped in the attack time are sweeps (actions to change position concerning the ground), throws (which occur after the transition time), guard passes, and chokes and locks submissions (Kirk et al., 2015; Coswig et al., 2018a). In addition, defenses happen when any of the athletes defend against the attack, as mentioned above, attempts (Kirk et al., 2015; Miarka et al., 2017a).

Low-intensity movement time: The actions grouped in the low-intensity movement time are activities without progression during the combat or grip adjustment and maintenance of a defensive position, that is, actions that do not contribute to changing the score or the superiority of the athlete over her opponent. Pauses were inserted in this movement context (Del Vecchio et al., 2011; Tornello et al., 2014; Barrientos et al., 2021).

### 2.3. Reliability testing

The reliability measures were observed through intra-observer testing procedures on BJJ time-motion data provided by one expert (i.e., >10 years of experience and with degrees in Physical Education). He analyzes BJJ matches with FRAMI software (Miarka et al., 2011). For intra-observer agreement - expert A analyzed 20 matches of BJJ athletes. Sequentially, expert A performed the intra-observer agreement, with the selection of the 10 BJJ matches (20 performance analyses) in a randomized order before repeating the time-motion analysis.

The reliability of time-motion variables analysis was examined using Cronbach’s Alpha Coefficient (CAC). From the frequency distribution for each variable, the following CAC values and strength of agreement classifications were used: Alpha values were described as excellent (0.93–0.94), strong (0.91–0.93), reliable (0.84–0.90), robust (0.81), reasonably high (0.76–0.95), high (0.73–0.95), good (0.71–0.91), relatively high (0.70–0.77), slightly low (0.68), reasonable (0.67–0.87), adequate (0.64–0.85), moderate (0.61–0.65), satisfactory (0.58–0.97), acceptable (0.45–0.98), sufficient (0.45–0.96), not satisfactory (0.4–0.55) and low (0.11). Statistical calculations were made using 22.0 SPSS software, and the significance level was set at *p* ≤ 0.05. The index and classification of Alpha values of BJJ time-motion indicators used in the present study are shown in Table 1.

**Table 1:** The reliability analysis and classification of CAC values of BJJ time-motion indicators.

BJJ combat time	Reliability	Classification	IC (95%) lower/Upper	Value–*p*
Approach	0.92	Excellent	0.89/0.96	≤0.01
Gripping	0.97	Excellent	0.96/0.99	≤0.01
Transition	0.93	Excellent	0.91/0.96	≤0.01
Guard	0.91	Strong	0.85/0.93	≤0.01
Side Control	0.95	Excellent	0.90/0.96	≤0.01
Mounting	0.89	Reliable	0.83/0.89	≤0.01
Attack	0.96	Excellent	0.89/0.96	≤0.01
Defense	0.98	Excellent	0.92/0.97	≤0.01
Low-intensity movement	0.89	Reliable	0.82/0.91	≤0.01
Total time	0.93	Strong	0.81/0.96	≤0.01

### 2.4. Statistical analysis

All analyses were conducted using SPSS 22.0 for Windows. Descriptive data are presented as median, mean [25th percentile; 75th percentile] values, and Kruskal Wallis followed by Bonferroni *post hoc* were used to compare time-motion seconds and frequencies between BJJ weight categories and the significance level of *p* ≤ 0.05 was used.

## 3. Results

Table 2 shows the time-motion analysis of the international female BJJ fighters in each combat phase, compared by weight category expressed in seconds.

**Table 2:** Time-motion analysis of the international female BJJ fighters in each combat phase, compared by weight category expressed in seconds.

Category	Approach	Gripping	Transition	Guard	Side control	Mounting	Attack	Defense	Low-intensity movement	Total time
Rooster (*n* = 8)	6.3 (5.2;11.1)	7.2^a^ (3.5;64.6)	14.0 (4.8;29.6)	104 (70.3;232.6)	9 (4.7;–)	261 (261;261)	76.2^pq^ (27.7;93.2)	30.6 (9.8;60.8)	72.1 (46.4;204.5)	408 (301.7;482)
Light feather (*n* = 18)	9.3 (6.0;14.6)	3.1^a^ (6.0;14.6)	1.5^g^ (1.2;4.3)	61 (28.0;96.2)	53.9 (31.5;173.1)	47 (15.7;193.6)	64.5^pq^ (17.3;111.4)	61.1 (19.9;102)	77.0 (29.6;242.5)	389.3 (305.8;444.9)
Feather (*n* = 122)	6.2 (3.7;11)	4.6^a^ (2.7d;13.8)	2.8^ho^ (1.6;4.8)	87.5 (38.8;169.9)	37.4 (19.6;66)	57.1 (19.2;128)	38.1^p^ (18.3;70)	30.5 (11.3;60.5)	93 (38.2;197.6)	359.6 (226.6;426.1)
Light (*n* = 84)	5.1 (3.0;13.5)	4.1^c^ (2.4;8.8)	2.1^hi^ (1.3;3.2)	111.8 (32.8;203.8)	60.5 (27.3;114.3)	26.9 (14.7;45.7)	42.7^p^ (15.5;4.6)	35.9 (13.8;73.7)	87.4 (25.4;204)	323 (210.3;386.9)
Middle (*n* = 74)	5.6 (2.8;11.1)	5.8^ae^ (2.3;13.5)	2.3^mn^ (1.6;4.4)	87.7 (41.8;130)	44 (28.9;69.7)	15.9 (11.0;55.6)	32.4^p^ (17.2; 69)	31.8 (13.8;51.2)	99.7 (40.8;187.4)	335.1 (241.8;417.5)
Medium heavy (*n* = 44)	6.1 (3.1;9.6)	3.3^b^ (1.8;8.3)	2.1 (1.6;2.9)	81.6 (22.3;185.5)	34.5 (17.6;51.3)	39.9 (13.4;207.3)	23.9 (7.2;64.7)	25.8 (12.0;102.9)	83.9 (41.1;159.9)	311.2 (89.9;556)
Heavy (*n* = 36)	6.1 (3.7;9.3)	4.5 (1.7f;29)	2.8 (1.7;29.0)	45.1 (18.9;152.3)	81.1 (37.5;136.3)	24.6 (17.3;70.3)	17.2^p^ (7.7;42.8)	16.8 (7.6;57.9)	86.0 (21.2;175.6)	314.8 (245.6;373)
Super heavy (*n* = 36)	4.8 (2.9;12.1)	3.1^abcdef^ (5.8;119.9)	2.1^j^ (1.4;3.5)	91.6 (41.7;145.7)	49.4 (29.3;108.8)	40 (22.1;65.6)	30.1 (14.0;61.4)	25.5 (10.3;43.9)	73.7 (37.8;176.7)	343.0 (287.2;495.5)
*H*	8.298	29.951	15.943	7.237	12.947	9.038	14.664	7.835	1.29	8.874
*P*	0.307	0.001	0.026	0.405	0.073	0.250	0.041	0.347	0.989	0.262

*p* = value of *p* (significance)/H - statistical test value. ^a^Significant difference between light feather and rooster. Feather. middle and super heavy. ^b^Significant difference between medium heavy and super heavy tracks. ^c^Significant difference between light and super heavy. ^d^Significant difference between light and super heavy. ^e^Significant difference between middle and super heavy. ^f^Significant difference between light and super heavy. ^g^Significant difference between light feather and rooster; ^h^Significant difference between light and feather. ^i^Significant difference between light and rooster; ^j^Significant difference between super heavy and rooster. ^l^Significant difference between medium heavy and rooster. ^m^Significant difference between middle and rooster. ^n^Significant difference between heavy and rooster. ^o^Significant difference between feather and rooster; ^p^Significant difference between heavy and middle. Light. feather. Light feather and rooster; ^q^Significant difference between medium heavy and light feather and rooster.

A significant difference was found in gripping moment (*K* = 29.951; *p* ≤ 0.001), transition (*H* = 15.943; *p* = 0.026) and attack time (*K* = 14.664; *p* = 0.041).

Table 3 demonstrates the post-hoc analysis of comparisons between weight divisions, in the combat phases (gripping, transition, and attack) among international female BJJ fighters.

**Table 3:** Time-motion post-hoc analysis of comparisons between weight divisions, used in the combat phases (gripping, transition and attack) among international female BJJ fighters.

Comparisons						
Group	Gripping		Transition		Attack	
	*H*	*p*	*H*	*p*	*H*	*p*
Light feather × Feather	−59.655	0.024	–	–	–	–
Light feather × Middle	−62.744	0.02	–	–	–	–
Light feather × Heavy	−64.373	0.029	–	–	–	–
Light feather × Rooster	102.286	0.016	112.101	0.002	–	–
Light feather × Super heavy	−132	0.001	–	–	–	–
Medium heavy × Rooster	–	–	102.155	0.003	–	–
Medium heavy × Super heavy	−94.031	0.001	–	–	–	–
Medium heavy × Light feather	–	–	–	–	57.593	0.052
Medium heavy × Rooster	–	–	–	–	85.673	0.034
Light × Rooster	–	–	106.177	0.001	–	–
Light × Feather	–	–	25.869	0.027	–	–
Light × Super heavy	−82.863	0.001	–	–	–	–
Feather × Super heavy	−72.343	0.001	–	–	–	–
Feather × Rooster	–	–	80.308	0.012	–	–
Middle × Super heavy	−69.254	0.001	–	–	–	–
Middle × Rooster	–	–	89.021	0.007	–	–
Heavy × Super heavy	−67.624	0.003	–	–	–	–
Super heavy × Rooster	–	–	104.205	0.004	–	–
Heavy × Rooster	–	–	85.456	0.013	109.536	0.008
Heavy × Middle	–	–	–	–	43.691	0.048
Heavy × Light	–	–	–	–	54.453	0.013
Heavy × Feather	–	–	–	–	55.753	0.007
Heavy × Light feather	–	–	–	–	81.455	0.007

*p* = value of *p* (significance)/H - statistical test value/Each row tests the null hypothesis where the group 1 and group 2 distributions are equal. Asymptotic (pairwise test) significances are displayed. The significance level is 0.05. Significance values were adjusted by Bonferroni correction for multiple tests.

Table 4 indicated the frequency of actions in each combat phase applied by international BJJ fighters in each phase of the combat. Compared according to weight category.

**Table 4:** Measures of the number of techniques applied by international BJJ fighters in each phase of the combat.

Time measures in combat phases, compared by weight category										
Category	Approach	Gripping	Transition	Guard	Side control	Mounting	Attack	Defense	Low–intensity movement	Total time
Rooster	2 (1;4)	1 (1;2)	3 (1;5^abcd^)	5 (2;10)	2 (1;–)	4 (4;4)	5 (3;6^d^)	3 (2;5^d^)	4 (2;5)	18 (12;30^bce^)
Light feather	1 (1;1)	1 (1;1ª)	1 (1;1ª)	3 (2;6)	3 (1;5)	2 (1;2)	3 (2.7)	4 (1;5^de^)	2 (1;5^d^)	17 (11;22)
Feather	1 (1;2)	1 (1;2^ab^)	1 (1;2^bcd^)	4 (2;6)	2 (1;4)	1 (1;2)	4 (2;6^bcde^)	3 (2;4^de^)	4 (2;7^d^)	16 (11;22^bcde^)
Light	1 (1;2)	1 (1;1^b^)	1 (1;1^b^)	3 (2;6)	1 (1;3)	1 (1;2)	3 (2;5)	2 (1;3d)	3 (1;6^d^)	14 (9;19)
Middle	1 (1;2)	1 (1;2^ab^)	1 (1;2^bcd^)	3 (2;6)	2 (2;4)	1 (1;2)	4 (6;6^d^)	3 (1;5^de^)	4 (2;7^de^)	18 (12;23^bcde^)
Medium heavy	1 (1;1)	1 (1;1)	1 (1;1^c^)	4 (1;7)	2 (1;4)	1 (1;2)	3 (1;5)	3 (1;5^d^)	4 (2;8^de^)	13 (7;20)
Heavy	1 (1;1)	1 (1;2)	1 (1;1^d^)	2 (1;3)	3 (1;4)	1 (1;1)	2 (1;5)	2 (2;3)	2 (1;4)	12 (8;15)
Super heavy	1 (1;2)	1 (1;3^abc^)	1 (1;1)	2 (1;4)	3 (2;3)	1 (1;2)	3 (1;4^e^)	2 (1;3)	2 (2;4)	15 (8;18)
*H*	10.006	16.092	21.738	13.459	6.014	4.758	15.671	24.418	22.336	30.994
*P*	0.188	0.024	0.003	0.062	0.538	0.69	0.028	0.001	0.002	0.001

Compared according to weight category. *p* = value of *p* (significance)/H - statistical test value. ^a^Significant difference between light feather. Rooster. feather. Middle and super heavy. ^b^Significant difference between light. Rooster. feather. Middle and super heavy. ^c^significant difference between medium heavy. Rooster. middle and super heavy. ^d^significant difference between heavy. Rooster. middle. Feather. light. Medium heavy and light feather. ^e^significant difference between super heavy. Feather. middle. Light feather. Medium heavy and rooster.

A significant difference in the frequency of techniques, and in the gripping time (*K* = 16.092; *p* = 0.024), transition (*K* = 21,738; *p* = 0.003), attack (*K* = 15.671; *p* = 0.028), defense (*K* = 24,418; *p* ≤ 0.001), movement with low-intensity (*K* = 22,336; *p* = 0.002) and in the total number of techniques (*K* = 30,994; *p* ≤ 0.001).

Table 5 shows a post-hoc analysis of comparisons between action frequencies used in the gripping, transition, attack, defense, and movement with low-intensity phases, according to the weight category used during the combat.

**Table 5:** Comparison of groups between international BJJ fighters’ techniques used in the grip phases.

Comparison												
Groups	Gripping		Transition		Attack		Defense		Low-intensity movement		Total Time	
	*H*	*p*	*H*	*p*	*H*	*p*	*H*	*p*	*H*	*p*	*H*	*p*
Light feather – Rooster	–	–	53.15	0.033	–	–	–	–	–	–	–	–
Light feather – Feather	−41.47	0.05	–	–	–	–	–	–	–	–	–	–
Light feather – Middle	−52.082	0.016	–	–	–	–	–	–	–	–	–	–
Light feather – Super Heavy	−56.223	0.016	–	–	–	–	–	–	–	–	–	–
Light – Rooster	–	–	54.875	0.012	–	–	–	–	–	–	91.693	0.042
Light – Feather	27.014	0.035	21.534	0.007	33.803	0.027	–	–	–	–	53.418	0.002
Light – Middle	−37.625	0.006	−23.514	0.012	–	–	–	–	–	–	−60.996	0.002
Light – Super heavy	−41.766	0.009	–	–	–	–	–	–	–	–	–	–
Medium heavy – Rooster	–	–	58.571	0.011	–	–	–	–	–	–	93.642	0.046
Medium heavy – Feather	–	–	25.231	0.016	40.455	0.036	–	–	–	–	55.366	0.01
Medium heavy – Middle	32.903	0.039	27.21	0.019	–	–	–	–	–	–	62.944	0.007
Medium heavy – Super heavy	−37.044	0.04	–	–	–	–	–	–	–	–	–	–
Heavy – Rooster	–	–	63.5	0.007	87.536	0.031	97.535	0.017	–	–	114.59	0.016
Heavy – Middle	–	–	32.139	0.01	47.025	0.032	79.762	0.001	79.609	0.001	83.893	0.001
Heavy – Feather	–	–	30.159	0.008	51.164	0.013	80.559	0.001	76.608	0.001	76.315	0.001
Heavy – Light	–	–	–	–	–	–	63.455	0.003	50.112	0.026	–	–
Heavy – Medium heavy	–	–	–	–	–	–	48.333	0.05	77.984	0.003	–	–
Heavy – Light feather	–	–	–	–	–	–	95.722	0.002	51.286	0.014	–	–
Super heavy – feather	–	–	–	–	43.027	0.049	53.32	0.013	–	–	70.176	0.002
Super heavy – Middle	–	–	–	–	–	–	52.523	0.023	54.286	0.014	77.754	0.002
Super heavy – Light feather	–	–	–	–	–	–	68.483	0.034	–	–	–	–
Super heavy – Medium heavy	–	–	–	–	–	–	–	–	52.661	0.043	–	–
Super heavy – Rooster	–	–	–	–	–	–	–	–	–	–	108.451	0.023

Transition, attack, defense, and the total of techniques according to the weight category used during the combat. *p* = value of *P* (significance)/H - statistical test value/Each row tests the null hypothesis where the group 1 and group 2 distributions are equal. Asymptotic (pairwise test) significances are displayed. The significance level is 0.05. Significance values were adjusted by Bonferroni correction for multiple tests.

## 4. Discussion

Findings with time-motion analysis allow a practical application with women because psychologists and coaches can use the results to prepare specific contextual demands of female matches, aiming for the specific category that the BJJ athlete competes (Amtmann, 2012; Coswig et al., 2018a; Brandt et al., 2021; Fernández et al., 2022). In this sense, some studies have specifically investigated time-motion analysis in female combat sports (Tornello et al., 2014; Andreato et al., 2015; Miarka et al., 2017b; Dal Bello et al., 2019). However, the studies have not analyzed the actions separate in the BJJ female weight divisions. The present research pointed out weight categories differences in female high-level BJJ match performance. Findings indicated that the Super heavyweight category had a shorter gripping time than other weight categories. In contrast, roosters had longer gripping, transition, and attack times and frequency than the light feather, middlers, and heavier weight categories.

The approach and gripping times are essential for efficiently applying guard and throw actions during BJJ matches (Coswig et al., 2018b; Williams et al., 2019). Coaches and psychologists consider that taking the initiative during BJJ combats is a factor that puts the athlete at an advantage (Faro et al., 2020; Brandt et al., 2021; Fernández et al., 2022). Grapplers who do not take the initiative with the handgrip tend to present complications dominating the opponent (Challis et al., 2015; Bahmani et al., 2019; Miarka et al., 2020a; Fernandes et al., 2022). Consequently, specific pieces of physical training are designed to approach and grip with speed and in the position that grips the opponent’s clothes (Andreato et al., 2015; Diaz-Lara et al., 2016; Ovretveit, 2018). In our study, roosters and super heavies presented critical dependence on gripping time, that specific information about the gripping is critical for coaches to incorporate into training to adjust and enhance the movements that result in specific tactical acquisition for each weight category (Sterkowicz-Przybycień et al., 2017; Miarka et al., 2020b). In past studies, BJJ high-level athletes presented similar behavior to Olympic judo athletes (Kuvacic et al., 2017; Miarka et al., 2017b; Barreto et al., 2019; Blach et al., 2021), with a lower time of approach and higher frequency of gripping. Our results indicated that differentiated attention should be directed to the rooster category. Since athletes in this category tend to make gripping defensive movements and a higher frequency of attacks, super heavy athletes have shorter gripping time - specific tactics could be elaborated in both categories for female athletes to anticipate the opponent’s behaviors. Since rooster and heavyweight athletes have a difference in the frequency and time of gripping moments, for each group of athletes, it would be recommended in the rooster category to pay greater attention to movements that may stop the gripping advantage of the opponents. At the same time, super heavy could increase sequential attacks, trying to induce the opponent to the groundwork combat.

In order to domain the opponent, it is essential to be incisive in the approach. Gripping, choose a gripping variation that allows advantage (i.e., attacks or guard moments) since generally, the female grapplers tend to apply the gripping arrangement that allows biomechanical advantage for the application of their favorite guard position or throws techniques (Dal Bello et al., 2019, Soto et al., 2020a,b). The past author observed that high-level female grappling athletes perform this moment with high speed and a lower amount of movements when compared to beginners and intermediates (Sterkowicz et al., 2016; Reale et al., 2018; Dopico-Calvo et al., 2022). Biomechanical aspects seemed to determine the type and preferred gripping since BJJ female athletes of lighter categories, possibly smaller in stature (Ovretveit, 2018), are the ones who least apply the back and leg gripping (Williams et al., 2019). Other highlighted aspects are the lower gripping and groundwork movement variation in all categories. In a previous study with female judo high-level athletes, Sterkowicz-Przybycień et al. (2017) found that female athletes tend to show single gripping and movement patterns - female grapplers prefer consistency and defensive movements, reducing the possibility of unpredictable adjustments. This fact could be partially confirmed in the present research and maybe the differential in vital phases of the BJJ combat, and that deserves more attention from the coaches and psychologists to make actions more unpredictable.

Reduced Attack times in the medium heavy; heavy groups are supported by the reportedly short timespan of throwing attempts and the ability to defend *via* gripping or counterattacks, similar to high-level judo female athletes (Sterkowicz-Przybycień et al., 2017). The present results established the relationship between skill level and attack velocity or guard establishment in female BJJ matches, where slower standing attacks were found in losing grappling female athletes at non-Olympic events (Miarka et al., 2016b). Furthermore, high-level female grappling athletes have been shown to prefer quick, effective throwing techniques (Sterkowicz et al., 2013), which affects effectiveness during groundwork actions (Miarka et al., 2016b; Williams et al., 2019).

Regarding the defensive combat phase, past findings indicated that female grappling winners at the Olympic Games had more frequent, shorter defensive actions than losers and all athletes in non-Olympic competitions (Miarka et al., 2016b). The observed within-athlete tactical variability in groundwork moment supports previous reports with judo that demonstrated visuomotor adjustments in response to domain attempts in talented grapplers, potentially due to enhanced anticipatory abilities (Dal Bello et al., 2019). During attacking actions, expert judo fighters spent a more significant percentage of their time fixating their gaze on their opponent’s lapel and face to detect the actions of their opponents. In contrast, beginners primarily fixated on more peripheral areas of their visual field, such as sleeves, hands, legs, and jacket skirts (Piras et al., 2014). The current study may have exhibited this adaptation by female BJJ weight categories to quickly identify critical moments, such as gripping and transition moments, to groundwork actions (i.e., Guard, Side Control, and Mounted) during competitive actions.

Pause and low-intensity moments are tactical ways to avoid a potential attack. During movements with low-intensity, low-intensity actions could result from attempts to avoid the opponent’s control. At the same time, pauses in BJJ are associated with penalties or specific situations (i.e., pause for illegal grip, communication with the referee, penalty for avoiding to fight, pause for injuries, cramps, and others). Furthermore, this seems to be a trend in other grappling fights. In judo, there was a considerable decrease in scores by the punishment at the 2012/2016/2020 Olympics Games (Sterkowicz et al., 2013; Miarka et al., 2016b; Barreto et al., 2019, 2022). Past research indicated 0.44 punishments/min during female judo combats in the 2012 Olympic Games versus 1.26 punishments/min during the 2008 Olympic Games and other international events (ranging from 1.46 to 2.17 punishments/min; Heinisch et al., 2013).

Regarding Guard, Side Control, and Mount moments, no effects of weight categories were observed in these groundwork actions; the reduction of weight dependence in groundwork combat may be responsible for increasing the time distributed to this combat phase in other grappling fights in the last years (Barreto et al., 2021, 2022; Miarka et al., 2022). Unlike present research with female BJJ athletes, the pause time during judo tournaments is likely related to the frequency of Groundwork combat actions because 20% of all attacking attempts have been shown to occur during the transition to or into the Groundwork phase (Heinisch et al., 2013). However, similar to the present results, the higher number of attacks in the transition and groundwork situations results in more techniques being applied (Kirk et al., 2015; Miarka et al., 2016b). Nonetheless, the frequency and duration of the low-intensity moment time allow a women grappler to place the opponent in a susceptible position during Guard, Side Control, and Mount moments.

### 4.1. Limitations

A possible limitation of BJJ time-motion analysis designated so far is the reliability of the data entry procedure (Avakian et al., 2021; Barrientos et al., 2021) and the present study indicated inter-expert and intra-expert measurement with different classifications index – the reliable, strong, and excellent (*r* range: 0.89–0.97) ability to reproduce the observed approach, gripping, transition, guard, side control, mounting, attack, defense, movement, and total time analysis. A challenge of the BJJ analysis process is subjectivity, where fast movements notated that have a certain degree of ambiguity may be captured differently by different analysts (Miarka et al., 2020b). Therefore, the observational-descriptive approach limits and extrapolates the present findings, which consist of different actions. Female BJJ athletes may also elicit different demands and physiological responses for specific action and combat phases (Del Vecchio et al., 2011; Sterkowicz-Przybycień et al., 2017).

### 4.2. Strengths

Objective female BJJ information allows for more evidence-based decisions during combats, reducing those based on speculation. Using evidence to give feedback to female combat athletes helps them to understand what they have done to be effective or ineffective, considering weight divisions. Training theories of grappling athletes stress that training programs should be comprised of three critical phases: preparatory, competition, and transition (Blumenstein et al., 2005). Physical, technical, tactical, and psychological preparations are present in each training phase and consider time-motion BJJ analysis to reproduce contextual demands (Del Vecchio et al., 2011, 2016). During the competition phase, which lasts between 4–8 months, female BJJ athletes should achieve their highest level of performance. Grapplers must pull it all together and reach their peak physically and psychologically (Blumenstein et al., 2005). In line, our results could help with five objectives in the competition phase (Lima et al., 2022): (a) to further improve the combat sport-specific abilities and psychological readiness, (b) to refine the skill level and technique, considering each weight category, (c) to enhance performance at the highest level in female grappling combats, (d) to improve tactics and strategies implemented in BJJ during training and competitions, and (e) to maintain general preparation, according to weight category necessities (Blumenstein et al., 2005).

### 4.3. Practical applications

The time-motion and behavior characteristics reported in this research, including the effort and pause ratio and the accumulated duration throughout a female BJJ match, provide data that can be used in both manipulations of training variables and periodization design, highlighting the competition phase with contextual training. Using the data provided in this research, BJJ coaches might choose to develop training sessions to improve specific behavior skills and BJJ-specific conditioning. Phase values for an athlete within a given category would guide the selection of sequential BJJ-specific work periods interspersed by “realistic” lower-effort behaviors (i.e., pause, movement, and approach phases) and higher intensities efforts (i.e., gripping, transition, guard, side control, mount, attack, and defense) periods. BJJ-specific work periods by weight category might include gripping, transition, and attack. Those phases are impacted by weight division, with longer gripping, transition, and attack times for roosters. As strength relative to lean body mass tends to be inversely related to body size, the lightest BJJ athletes may exhibit greater muscular endurance and anaerobic power-to-weight ratio (Del Vecchio et al., 2016).

In contrast, light feather, feather, light, and middle categories had longer attack times than heavy athletes. In the heaviest categories, BJJ display the highest body fat percentages and the lowest relative strength compared to other divisions (Del Vecchio et al., 2016). Sterkowicz-Przybycień et al. (2017) showed a higher frequency of specific throwing techniques in male and female lighter-weight judo athletes compared to heavier-weight divisions using a biomechanical approach to classify judo throws. Thus, unique phenotypes and physical qualities among female BJJ athletes in the lowest and highest categories may have precluded potential differences in combat’s gripping, transition, and attack phases.

### 4.4. Conclusion

The pioneering research compared continuous female high-level BJJ actions in time and frequency, considering weight categories. Our principal findings showed that the Super heavyweight category had a shorter gripping time than other weight categories. In contrast, roosters had a longer gripping, transition, and attack times and frequency than the light feather, middlers, and heavier weight categories. In summary, the roosters’ category demonstrated more extended actions. There is a trend to equalize middle and lightweight categories in high-intensity actions. Future psychological studies might consider other technical, tactical, and emotional analyses associated with performance.

## Data availability statement

The original contributions presented in the study are included in the article/supplementary material, further inquiries can be directed to the corresponding authors.

## Ethics statement

Ethical review and approval was not required for the study on human participants in accordance with the local legislation and institutional requirements. Written informed consent from the [patients/ participants OR patients/participants legal guardian/next of kin] was not required to participate in this study in accordance with the national legislation and the institutional requirements.

## Author contributions

All authors listed have made a substantial, direct, and intellectual contribution to the work and approved it for publication.

## Conflict of interest

The authors declare that the research was conducted in the absence of any commercial or financial relationships that could be construed as a potential conflict of interest.

## Publisher’s note

All claims expressed in this article are solely those of the authors and do not necessarily represent those of their affiliated organizations, or those of the publisher, the editors and the reviewers. Any product that may be evaluated in this article, or claim that may be made by its manufacturer, is not guaranteed or endorsed by the publisher.

## Footnotes

^1^ https://www.youtube.com/user/ibjjf
