# Corrigendum: Effects of weight divisions in time-motion of female high-level Brazilian Jiu-jitsu combat behaviors

**DOI:** 10.3389/fpsyg.2023.1196198

**Published:** 2023-04-17

**Authors:** Marco Antonio Ferreira dos Santos, Dany Alexis Sobarzo Soto, Michele Andrade de Brito, Ciro José Brito, Esteban Aedo-Muñoz, Maamer Slimani, Nicola L. Bragazzi, Hela Znazen, Bianca Miarka

**Affiliations:** ^1^Laboratory of Psychophysiology and Performance in Sports and Combats, Postgraduate Program in Physical Education, Federal University of Rio de Janeiro, Rio de Janeiro, Brazil; ^2^Escuela de Kinesiología, Facultad de Salud, Universidad Santo Tomás, Puerto Montt, Chile; ^3^Postgraduate Program in Physical Education, Federal University of Juiz de Fora, Governador Valadares, Brazil; ^4^Departamento de Educación Física, Deportes y Recreación, Facultad de Artes y Educación Física, Universidad Metropolitana de Ciencias de la Educación, Santiago, Chile; ^5^Department of Health Sciences (DISSAL), University of Genoa, Genoa, Italy; ^6^Department of Mathematics and Statistics, Laboratory for Industrial and Applied Mathematics (LIAM), York University, Toronto, ON, Canada; ^7^Department of Physical Education and Sport, College of Education, Taif University, Taif, Saudi Arabia

**Keywords:** sports psychology, technical-tactical analysis, task performance and analysis, judo, martial arts

In the published article an Acknowledgments statement was mistakenly excluded. The correct Acknowledgments statement appears below:

